# PCTFPeval: a web tool for benchmarking newly developed algorithms for predicting cooperative transcription factor pairs in yeast

**DOI:** 10.1186/1471-2105-16-S18-S2

**Published:** 2015-12-09

**Authors:** Fu-Jou Lai, Hong-Tsun Chang, Wei-Sheng Wu

**Affiliations:** 1 Department of Electrical Engineering, National Cheng Kung University, Tainan, Taiwan

**Keywords:** Web tool, Cooperative transcription factor pairs, Performance index, Performance comparison, Algorithm

## Abstract

**Background:**

Computational identification of cooperative transcription factor (TF) pairs helps understand the combinatorial regulation of gene expression in eukaryotic cells. Many advanced algorithms have been proposed to predict cooperative TF pairs in yeast. However, it is still difficult to conduct a comprehensive and objective performance comparison of different algorithms because of lacking sufficient performance indices and adequate overall performance scores. To solve this problem, in our previous study (published in BMC Systems Biology 2014), we adopted/proposed eight performance indices and designed two overall performance scores to compare the performance of 14 existing algorithms for predicting cooperative TF pairs in yeast. Most importantly, our performance comparison framework can be applied to comprehensively and objectively evaluate the performance of a newly developed algorithm. However, to use our framework, researchers have to put a lot of effort to construct it first. To save researchers time and effort, here we develop a web tool to implement our performance comparison framework, featuring fast data processing, a comprehensive performance comparison and an easy-to-use web interface.

**Results:**

The developed tool is called PCTFPeval (Predicted Cooperative TF Pair evaluator), written in PHP and Python programming languages. The friendly web interface allows users to input a list of predicted cooperative TF pairs from their algorithm and select (i) the compared algorithms among the 15 existing algorithms, (ii) the performance indices among the eight existing indices, and (iii) the overall performance scores from two possible choices. The comprehensive performance comparison results are then generated in tens of seconds and shown as both bar charts and tables. The original comparison results of each compared algorithm and each selected performance index can be downloaded as text files for further analyses.

**Conclusions:**

Allowing users to select eight existing performance indices and 15 existing algorithms for comparison, our web tool benefits researchers who are eager to comprehensively and objectively evaluate the performance of their newly developed algorithm. Thus, our tool greatly expedites the progress in the research of computational identification of cooperative TF pairs.

## Background

Understanding combinatorial or cooperative transcriptional regulation by two or more transcription factors (TFs) has become an important research topic in the recent decade. Researchers have studied and modelled various types of TF-TF interactions which contribute to positive or negative synergy in regulating genes [1-3]. Attributing to the availability of various kinds of genome-wide datasets (e.g. gene expression data, ChIP-chip data, TF binding site motifs, protein-protein interaction data and TF knockout data), researchers continued developing advanced algorithms to predict cooperative TF pairs. Some algorithms only utilized ChIP-chip data [3-6] or gene expression data [7], and the others integrated multiple data sources [8-17].

Since different algorithms integrated different data sources, used different rationales and predicted distinct lists of cooperative TF pairs, it is hard to tell which one is the best. Typically, researchers only compared their algorithm with a few existing algorithms using a few performance indices (see Table 1) and claimed their algorithm to be the best one. However, this kind of comparison is incomplete and subjective [18]. A comprehensive and objective performance comparison framework is urgently needed.

**Table 1:** The numbers of the compared algorithms, the performance indices, and the predicted cooperative TF pairs (PCTFPs) for each of the 15 existing algorithms.

Algorithm	# of existing algorithms used for performance comparison in their paper	# of indices used for performance evaluation in their paper	# of PCTFPs
Banerjee and Zhang (NAR 2003) [8]	0	1	31

Harbison et al. (Nature 2004) [4]	0	0	94

Nagamine et al. (NAR 2005) [9]	0	1	24

Tsai et al. (PNAS 2005) [10]	0	1	18

Chang et al. (Bioinformatics 2006) [11]	2	1	55

He et al. (IEEE GCCW 2006) [12]	2	1	30

Yu et al. (NAR 2006) [5]	0	1	300

Wang J (JBI 2007) [13]	3	1	14

Elati et al. (Bioinformatics 2007) [7]	4	1	20

Datta and Zhao (Bioinformatics 2008) [6]	3	1	25

Chuang et al. (BMC Bioinformatics 2009) [14]	4	2	13

Wang Y et al. (NAR 2009) [15]	5	2	159

Yang et al. (Cell Research 2010) [16]	3	1	186

Chen et al. (Bioinformatics 2012) [3]	2	2	221

Lai et al. (BMC Systems Biology 2014) [17]	11	3	27

To meet this need, in our previous study [19], we proposed/adopted eight performance indices to compare the performance of 14 existing algorithms. Our results showed that the performance of an algorithm varies widely across different performance indices, implying that researchers may make a biased conclusion based on only a few performance indices. Therefore, in order to conduct a comprehensive and objective performance comparison, we designed two overall performance scores to summarize the comparison results of the eight performance indices.

Most importantly, our performance comparison framework can be applied to comprehensively and objectively evaluate the performance of a newly developed algorithm. Therefore, researchers who develop a new algorithm definitely would like to use our performance comparison framework to quickly evaluate the prediction performance in order for improvement when needed. However, to use our framework, researchers have to put a lot of effort to construct it first. Constructing our framework involves collecting and processing multiple genome-wide datasets from the public domain, collecting the lists of the predicted cooperative TF pairs from 15 existing algorithms in the literature, and writing a lot of codes to implement the eight performance indices. To save researchers time and effort, here we develop a web tool called PCTFPeval (Predicted Cooperative TF Pair evaluator) to implement our performance comparison framework, featuring fast data processing, a comprehensive performance comparison and an easy-to-use web interface. Constructing PCTFPeval is not a daunting task for us since we already have many experiences in developing databases and web tools [20-26].

## Implementation

### Fifteen existing algorithms used for performance comparison

Our tool provides 15 existing algorithms for users to conduct a performance comparison. As far as we know, this is the most comprehensive collection of the existing algorithms whose lists of the predicted cooperative TF pairs in yeast are available. The numbers of the predicted cooperative TF pairs from different algorithms vary widely, ranging from 13 to 300 (see Table 1).

### Eight existing performance indices used for performance evaluation

Our tool implements eight existing performance indices for users to evaluate the performance of an algorithm for predicting cooperative TF pairs in yeast. As far as we know, this is the most comprehensive collection of the existing performance indices. These eight performance indices can be divided into two types: TF-based indices and target gene based (TG-based) indices. Each type has four indices and different indices utilize different data sources and rationales (see Table 2).

**Table 2:** The eight performance indices implemented in our tool

Performance index type	Index	Data sources used	Rationale
TF-based	Index1	Yeast physical PPI data from BioGRID database [27]	Measure the overlap significance of the physical PPI partners of a PCTFP*
	Index2	Yeast physical PPI data from BioGRID database [27]	Measure the shortest path length of a PCTFP in the physical PPI network
	
	Index3	Yang et al.'s functional similarity scores of any two yeast genes [28]	Measure the functional similarity of a PCTFP
	
	Index4	Yang et al.'s high-quality benchmark set of 27 cooperative TF pairs in yeast [16]	Measure the overlap significance of the list of PCTFPs from an algorithm and the benchmark set of 27 cooperative TF pairs

TG-based	Index5	Balaji et al.'s co-regulatory coefficient dataset of 3459 TF pairs in yeast [29]	Measure the co-regulatory coefficient of a PCTFP
	
	Index6	Co-expression scores of any two yeast genes from SPELL database [30] and TF-gene documented regulation data from YEASTRACT database [31]	Measure the expression coherence of a PCTFP's common target genes
	
	Index7	Yang et al.'s functional similarity scores of any two yeast genes [28] and TF-gene documented regulation data from YEASTRACT database [31]	Measure the functional coherence of a PCTFP's common target genes
	
	Index8	Yeast physical PPI data from BioGRID database [27] and TF-gene documented regulation data from YEASTRACT database [31]	Measure the physical PPI coherence of a PCTFP's common target genes

*PCTFP is the abbreviation for predicted cooperative TF pair.

Different indices utilizes different data sources and rationales. See our previous study [19] for the details about the mathematics of these eight performance indices.

### Two existing overall performance scores used for representing the comprehensive performance comparison results

Our tool implements two existing overall performance scores [19] to summarize the comparison results of the selected performance indices. The first one is called the comprehensive ranking score defined as the sum of the rankings in the selected performance indices [19]. The ranking of an algorithm in an index is *k *if its performance ranks *#k *among all the compared algorithms in that index. For example, the ranking of the best performing algorithm is 1. Therefore, the smaller the comprehensive ranking score, the better the overall performance of an algorithm.

The second overall performance score is called the comprehensive normalized score (*CNS*) defined as the sum of the normalized scores in the selected performance indices [19]. The *CNS *of the algorithm *i *is calculated as follows:

$$CNS\left(i\right)=\overset{\text{L}}{\underset{j=1}{\sum }}NS_{j}\left(i\right)=\overset{\text{L}}{\underset{j=1}{\sum }}\left(\frac{OS_{j}\left(i\right)}{\text{max}\left(OS_{j}\left(1\right),OS_{j}\left(2\right),...,OS_{j}\left(n\right)\right)}\right)$$

where $NS_{j}\left(i\right)$ and $OS_{j}\left(i\right)$ is the normalized score and the original score of the algorithm *i *calculated using the index *j*, respectively; *n *is the number of the algorithms being compared; *L *is the number of the selected indices. Note that $0\leq NS_{j}\left(i\right)\leq 1$ and $NS_{j}\left(i\right)=1$ if and only if the algorithm *i *is the best performing algorithm in the index *j *(i.e. it has the highest original score calculated using the index *j*). The larger the *CNS*, the better the performance of an algorithm.

## Results and discussion

### Usage

**Figure 1:**
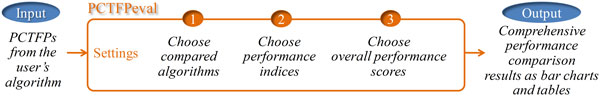
**The conceptual flowchart of our tool**. The flowchart shows the procedure of using our tool to conduct a comprehensive performance comparison of the user's algorithm to many existing algorithms using various performance indices.

The conceptual flowchart of our tool is shown in Figure 1. The friendly web interface allows users to input a list of the predicted cooperative TF pairs from their algorithm. Then three kinds of settings of our tool have to be specified. First, users have to choose the compared algorithms among the 15 existing algorithms. Second, users have to choose the performance indices among the eight existing indices. Finally, users have to choose the overall performance scores from the comprehensive ranking score and the comprehensive normalized score. After the submission, our tool conducts a comprehensive performance comparison of the user's algorithm to the compared algorithms using the selected performance indices. The comprehensive performance comparison results are then generated in tens of seconds and shown as both bar charts and tables.

### Case study

**Figure 2:**
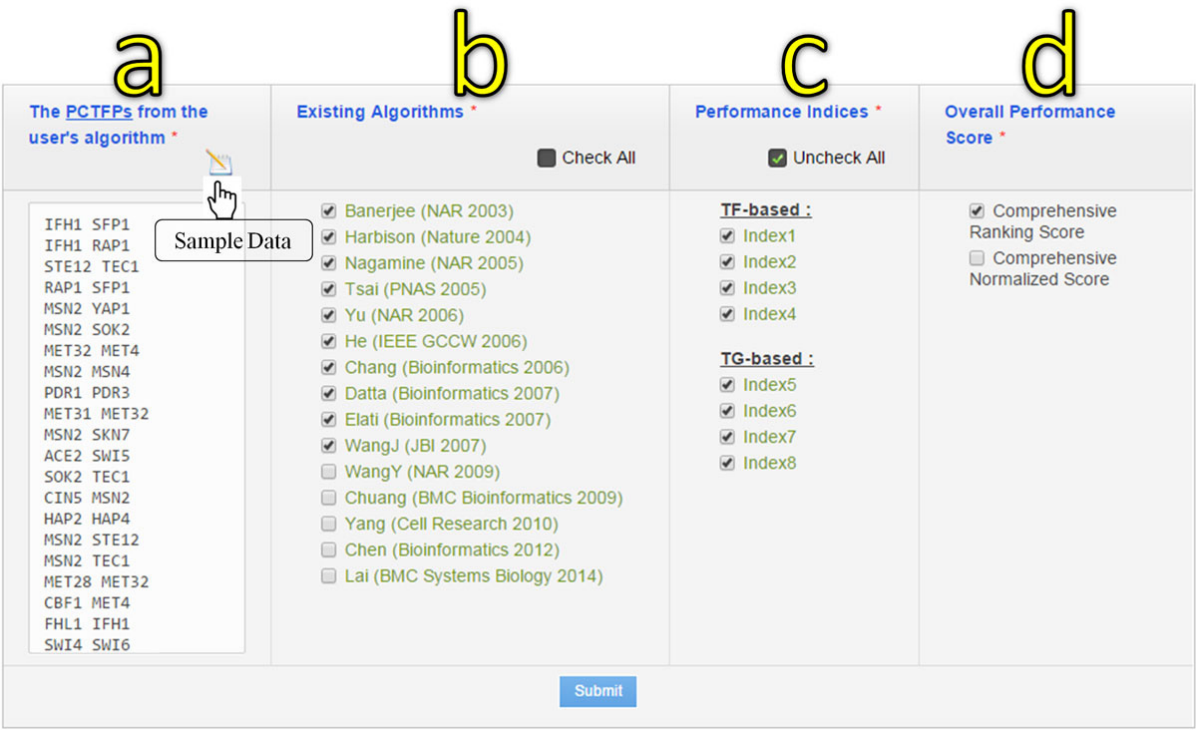
**The input and three settings of our tool**. To use our tool, users have to (a) input a list of the predicted cooperative TF pairs (PCTFPs) from their algorithm and select (b) the compared algorithms among the 15 existing algorithms, (c) the performance indices among the eight existing indices, and (d) the overall performance scores from the comprehensive ranking score and the comprehensive normalized score.

**Figure 3:**
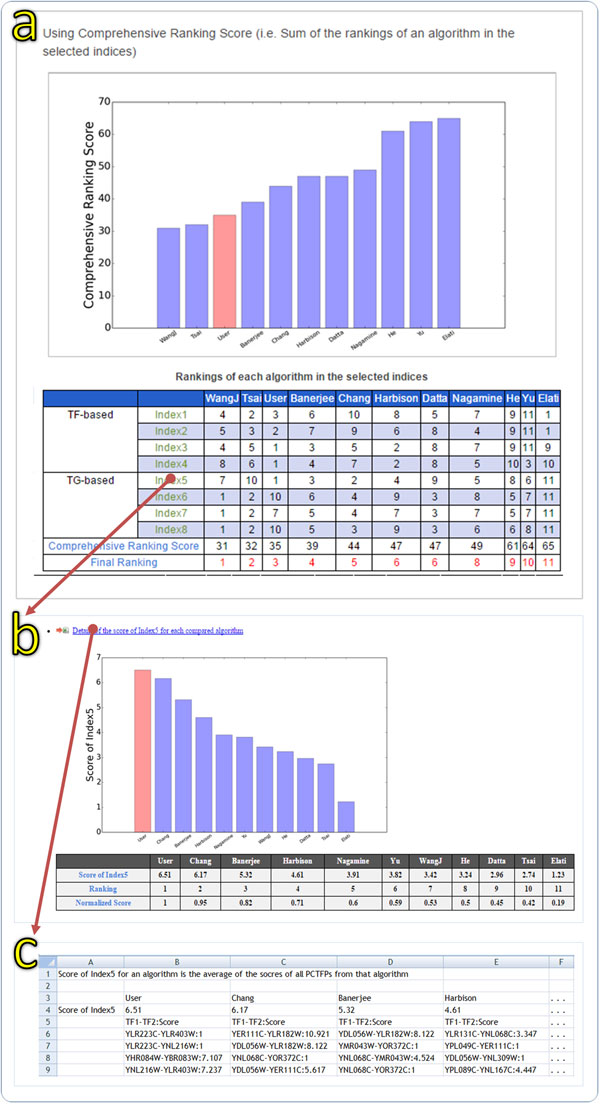
**The output of our tool**. Here we input the sample data (a list of 40 TF pairs) as a list of the predicted cooperative TF pairs (PCTFPs) from a user's algorithm and select 10 existing algorithms, eight performance indices, and the comprehensive ranking score as the overall performance score. (a) The comprehensive performance comparison results are shown as a bar chart and a table. It can be seen that the overall performance of the user's algorithm ranks three among all the 11 algorithms being compared. (b) When clicking the hyperlink of "Index5", users will get the performance comparison results (shown as both a bar chart and a table) using only the index 5. It can be seen that the user's algorithm is the best performing algorithm in the index 5. (c) When clicking the hyperlink of "Details of the score of Index5 for each compared algorithm", users will get a text file containing the original scores (calculated using the index 5) of all PCTFPs of each algorithm being compared.

In our tool, a list of 40 TF pairs is provided as a sample data. For demonstration purpose, we regard the sample data as the list of the predicted cooperative TF pairs from a new algorithm and would like to conduct a comprehensive performance comparison of this new algorithm to the various existing algorithms using our tool. As shown in Figure 2, users input the sample data to our tool and select (i) 10 existing algorithms for comparison, (ii) eight performance indices for evaluation, and (iii) the comprehensive ranking score as the overall performance score. After the submission, the comprehensive comparison results are generated and shown as both bar charts and tables (see Figure 3). It can be seen that the new algorithm performs well in the first five performance indices but performs worse in the last three performance indices. The overall performance of the new algorithm ranks three among all the 11 algorithms being compared. Getting the comprehensive comparison results from our tool, researchers immediately know that there is still room to improve the performance of their new algorithm.

## Conclusions

Knowing the cooperative TFs is crucial for understanding the combinatorial regulation of gene expression in eukaryotic cells. This is why the computational identification of cooperative TF pairs has become a hot research topic. Researchers will keep developing new algorithms. Using our tool, researchers can quickly conduct a comprehensive and objective performance comparison of their new algorithm to the various existing algorithms. If the performance of their new algorithm is not satisfactory, researchers can modify their algorithm and use our tool again to see if the performance is improved. Therefore, having our tool in hand, researchers can now totally focus on designing new algorithms and need not worry about how to comprehensively and objectively evaluate the performance of their new algorithms. In conclusion, our tool can greatly expedite the progress in this research topic.

## Availability and requirements

Project name: PCTFPeval

Project home page: http://cosbi.ee.ncku.edu.tw/PCTFPeval/

Operating system(s): platform independent.

Programming language: PHP, Python and Javascript.

Other requirements: Internet connection.

License: none required.

Any restrictions to use by non-academics: no restriction.

## Competing interests

The authors declare that they have no competing interests.

## Authors' contributions

WSW conceived the research topic and provided essential guidance. WSW and FJL developed the method and wrote the manuscript. FJL collected and processed all the genome-wide datasets used in this tool. HTC constructed the web interface of this tool. All authors read, edited and approved the final manuscript.

## Declarations

The publication of this paper was funded by National Cheng Kung University and Ministry of Science and Technology of Taiwan MOST-103-2221-E-006 -174 -MY2.

This article has been published as part of *BMC Bioinformatics *Volume 16 Supplement 18, 2015: Joint 26th Genome Informatics Workshop and 14th International Conference on Bioinformatics: Bioinformatics. The full contents of the supplement are available online at http://www.biomedcentral.com/bmcbioinformatics/supplements/16/S18.

## References

[B1] Miller JA Widom J Collaborative competition mechanism for gene activation in vivo Mol Biol Cell 2003 23 5 1623 1632 10.1128/MCB.23.5.1623-1632.2003 PMC151720 12588982

[B2] Tanay A Extensive low-affinity transcriptional interactions in the yeast genome Genome Res 2006 16 962 972 16809671 10.1101/gr.5113606 PMC1524868

[B3] Chen MJ Chou LC Hsieh TT Lee DD Liu KW Yu CY Oyang YJ Tsai HK Chen CY De novo motif discovery facilitates identification of interactions between transcription factors in Saccharomyces cerevisiae Bioinformatics 2012 28 5 701 708 22238267 10.1093/bioinformatics/bts002

[B4] Harbison CT Gordon DB Lee TI Rinaldi NJ Macisaac KD Danford TW Hannett NM Tagne JB Reynolds DB Yoo J Jennings EG Zeitlinger J Pokholok DK Kellis M Rolfe PA Takusagawa KT Lander ES Gifford DK Fraenkel E Young RA Transcriptional regulatory code of a eukaryotic genome Nature 2004 431 7004 99 104 15343339 10.1038/nature02800 PMC3006441

[B5] Yu X Lin J Masuda T Esumi N Zack DJ Qian J Genome-wide prediction and characterization of interactions between transcription factors in Saccharomyces cerevisiae Nucleic Acids Res 2006 34 17 917 927 16464824 10.1093/nar/gkj487 PMC1361616

[B6] Datta D Zhao H Statistical methods to infer cooperative binding among transcription factors in Saccharomyces cerevisiae Bioinformatics 2008 24 545 552 17989095 10.1093/bioinformatics/btm523

[B7] Elati M Neuvial P Bolotin-Fukuhara M Barillot E Radvanyi F Rouveirol C LICORN: learning cooperative regulation networks from gene expression data Bioinformatics 2007 23 18 2407 2414 17720703 10.1093/bioinformatics/btm352

[B8] Banerjee N Zhang MQ Identifying cooperativity among transcription factors controlling the cell cycle in yeast Nucleic Acids Res 2003 31 7024 7031 14627835 10.1093/nar/gkg894 PMC290262

[B9] Nagamine N Kawada Y Sakakibara Y Identifying cooperative transcriptional regulations using protein-protein interactions Nucleic Acids Res 2005 33 4828 4837 16126847 10.1093/nar/gki793 PMC1192832

[B10] Tsai HK Lu HHS Li WH Statistical methods for identifying yeast cell cycle transcription factors Proc Natl Acad Sci USA 2005 102 13532 13537 16157877 10.1073/pnas.0505874102 PMC1224643

[B11] Chang YH Wang YC Chen BS Identification of transcription factor cooperativity via stochastic system model Bioinformatics 2006 22 18 2276 2282 16844711 10.1093/bioinformatics/btl380

[B12] He D Zhou D Zhou Y Xiao N, Buyya R, Liu Y, Yang G Identifying synergistic transcriptional factors involved in the yeast cell cycle using Microarray and ChIP-chip data Proceedings of the Fifth International Conference on Grid and Cooperative Computing Workshops:21-23 October 2006; Hunan 2006 Los Alamitos: IEEE Computer Society 357 360

[B13] Wang J A new framework for identifying combinatorial regulation of transcription factors: a case study of the yeast cell cycle J Biomedical Informatics 2007 40 6 707 725 10.1016/j.jbi.2007.02.003 17418646

[B14] Chuang CL Hung K Chen CM Shieh GS Uncovering transcriptional interactions via an adaptive fuzzy logic approach BMC Bioinformatics 2009 10 400 19961622 10.1186/1471-2105-10-400 PMC2797023

[B15] Wang Y Zhang XS Xia Y Predicting eukaryotic transcriptional cooperativity by Bayesian network integration of genome-wide data Nucleic Acids Res 2009 37 18 5943 5958 19661283 10.1093/nar/gkp625 PMC2764433

[B16] Yang Y Zhang Z Li Y Zhu XG Liu Q Identifying cooperative transcription factors by combining ChIP-chip data and knockout data Cell Res 2010 20 11 1276 1278 20975739 10.1038/cr.2010.146

[B17] Lai FJ Jhu MH Chiu CC Huang YM Wu WS Identifying cooperative transcription factors in yeast using multiple data sources BMC Systems Biology 2014 8 Suppl 5 S2 25559499 10.1186/1752-0509-8-S5-S2 PMC4305981

[B18] Norel R Rice JJ Stolovitzky G The self-assessment trap: can we all be better than average? Mol Syst Biol 2011 7 537 21988833 10.1038/msb.2011.70 PMC3261704

[B19] Lai FJ Chang HT Huang YM Wu WS A comprehensive performance evaluation on the prediction results of existing cooperative transcription factors identification algorithms BMC Systems Biology 2014 8 Suppl 4 S9 25521604 10.1186/1752-0509-8-S4-S9 PMC4290732

[B20] Chang DTH Huang CY Wu CY Wu WS YPA: an integrated repository of promoter features in Saccharomyces cerevisiae Nucleic Acids Res 2011 39 1 D647 D652 21045055 10.1093/nar/gkq1086 PMC3013683

[B21] Chang DTH Li WS Bai YH Wu WS YGA: identifying distinct biological features between yeast gene sets Gene 2012 518 1 26 34 23266802 10.1016/j.gene.2012.11.089

[B22] Chiu CC Chan SY Wang CC Wu WS Missing value imputation for microarray data: a comprehensive comparison study and a web tool BMC Syst Biol 2013 7 Suppl 6 S12 24565220 10.1186/1752-0509-7-S6-S12 PMC4028811

[B23] Yang TH Wang CC Wang YC Wu WS YTRP: a repository for yeast transcriptional regulatory pathways Database 2014 bau014 24608172 10.1093/database/bau014 PMC3948430

[B24] Yang TH Chang HT Hsiao ESL Sun JL Wang CC Wu HY Liao PC Wu WS iPhos: toolkit to streamline the alkaline phosphatase assisted comprehensive LC-MS phosphorproteome investigation BMC Bioinformatics 2014 15 Suppl 16 S10 25521246 10.1186/1471-2105-15-S16-S10 PMC4290636

[B25] Yang TH Wang CC Hung PC Wu WS cisMEP: an integrated repository of genomic epigenetic profiles and cis-regulatory modules in Drosophila BMC Syst Biol 2014 8 Suppl 4 S8 25521507 10.1186/1752-0509-8-S4-S8 PMC4290730

[B26] Hung PC Yang TH Liaw HJ Wu WS YNA: an integrative gene mining platform for studying chromatin structure and its regulation in Yeast BMC Genomics 2014 15 Suppl 9 S5 25522035 10.1186/1471-2164-15-S9-S5 PMC4290617

[B27] Stark C Breitkreutz BJ Chatr-Aryamontri A Boucher L Oughtred R Livstone MS Nixon J Van Auken K Wang X Shi X Reguly T Rust JM Winter A Dolinski K Tyers M The BioGRID Interaction Database: 2011 update Nucleic Acids Res 2011 39 Database issue D698 D704 21071413 10.1093/nar/gkq1116 PMC3013707

[B28] Yang H Nepusz T Paccanaro A Improving GO semantic similarity measures using download random walks Bioinformatics 2012 28 10 1383 1389 22522134 10.1093/bioinformatics/bts129

[B29] Balaji S Babu MM Iyer LM Luscombe NM Aravind L Comprehensive analysis of combinatorial regulation using the transcriptional regulatory network of yeast J Mol Biol 2006 360 1 213 227 16762362 10.1016/j.jmb.2006.04.029

[B30] Hibbs MA Hess DC Myers CL Huttenhower C Li K Troyanskaya OG Exploring the functional landscape of gene expression: directed search of large microarray compendia Bioinformatics 2007 23 20 2692 2699 17724061 10.1093/bioinformatics/btm403

[B31] Abdulrehman D Monteiro PT Teixeira MC Mira NP Lourenço AB dos Santos SC Cabrito TR Francisco AP Madeira SC Aires RS Oliveira AL Sá-Correia I Freitas AT YEASTRACT: providing a programmatic access to curated transcriptional regulatory associations in Saccharomyces cerevisiae through a web services interface Nucleic Acids Res 2011 39 Database issue D136 D140 20972212 10.1093/nar/gkq964 PMC3013800

